# Supplementary material for: The emergence of circadian timekeeping in the intestine
Source: Nat Commun. 2024 Feb 27;15:1788. doi: 10.1038/s41467-024-45942-4 (PMC10899604; doi:10.1038/s41467-024-45942-4)
Supplement: Supplementary file 1 — Supplementary Information [file 41467_2024_45942_MOESM1_ESM.pdf]

Supplementary Information File

Contents

Supplementary Table 1. Fly Strains, by figure number.

Statistics, by figure number.

Supplementary Figure 1. Correlation Matrices for Figure 3C-D

Supplementary Figure 2. Related to Figure 1. *per*^{01} and *CantonS* Flies Develop at the Same Rate.

Supplementary Figure 3. Related to Figure 1. Circadian Clock Activity is present in Adults.

Supplementary Figure 4. Related to Figure 2. Clk/cyc Activity is Rhythmic at Adult Day 4 in ECs.

Supplementary Figure 5. Related to Figure 3. Adult Cells Express Circadian Clock Genes at Higher Levels.

Supplementary Figure 6. Related to Figure 4-6. Testing the Correlation of Circadian Clock Expression and ISC Differentiation.

Supplementary Figure 7. Related to Figure 7. Fasting Does Not Disrupt Established Rhythms.

Supplementary Data 1. Related to Figure 2. Early Pupa Intestine Raw Counts.

Supplementary Data 2. Related to Figure 2. Immature Adult Intestine Raw Counts.

Supplementary Data 3. Related to Figure 2. Mature Adult Intestine Raw Counts.

Supplementary Data 4. Related to Figure 3-6. Cluster Markers, determined by Seurat using ROC analysis with a logFC threshold of 0.25 and minimum percentage of cells of 0.25.

Supplementary Data 5. Related to Figure 5. *Clock*^{TIM} RNAi Screen.

References

Supplementary Table 1: Fly Strains

Figure	Panel	Genotype
**1**	B	*6xSTAT-dGFP(II)*
**1**	D	*Myo1A-Gal4/+;UAS-mCherry/Clock*^{PER} (images)
**1**	D	*Clock*^{PER}*(II)*
**1**	E	*Clock*^{PER}*(II)*
**1**	F	*Clock*^{TIM}*(III)*
**1**	G	*Clock*^{TIM}*(III)*
**1**	H	*CantonS*
**2**	A	*CantonS*
**2**	B	*Clock*^{PER}*(II)*
**2**	C-D	*Clock*^{TIM}*(III)*
**2**	E	*per-AID-eGFP (I)*
**3**	A-D	*CantonS*
**4**	A-C	*CantonS*
**5**	A	*esg-Gal4/Clock*^{TIM}*;tubGal80*^{TS}*/RNAi or esg-Gal4/RNAi;tubGal80*^{TS}*/ Clock*^{TIM}
**5**	B	For gce, Eip78C, Hnf4 #1, Hr78 #1, Eip75B #1 and #2: *esg-Gal4,UAS-mCherry,Clock*^{TIM}*/+;tubGal80*^{TS}*/overexpression*<br>For Hr78 #2, met, Hnf4 #1: *esg-Gal4,UAS-mCherry,Clock*^{TIM}*/overexpression;tubGal80*^{TS}*/+*<br>Control (crossed to *CantonS*): *esg-Gal4,UAS-mCherry,Clock*^{TIM}*/+;tubGal80*^{TS}*/+*
**5**	C	*Myo1A-Gal4/Clock*^{TIM}*;tubGal80TS/RNAi or Myo1A-Gal4/RNAi;tubGal80TS/ Clock*^{TIM}
**6**	A-C	*CantonS*
**6**	D	*act>y+>Gal4,UAS-CD2/hs-Flp;Clock*^{TIM}*/+*
**6**	E	*esg-Gal4/Clock*^{PER}*;Su(H)GBE-Gal80,tubGal80*^{TS}*/UAS-mCherry*
**6**	F	*Su(H)-Gal4/Clock*^{PER}*;UAS-mCherry/tubGal80TS*
**6**	G	*Myo1A-Gal4/Clock*^{PER}*;tubGa80*^{TS}*/UAS-mCherry*
**7**	A	*Clock*^{TIM}*(III)*
**7**	B-C	*Clock*^{TIM}*;cry*^{01}
**S2**	A-B	*CantonS* and *per*^{01}
**S2**	C-E	*Clock*^{PER}*(II)*
**S2**	F-G	*Clock*^{TIM}*(III)*
**S3**	A	*Clock*^{PER}*(II)*
**S3**	B	*cry-GFP(II)*
**S3**	C	*per-AID-eGFP(I)*
**S3**	D	*Clock*^{TIM}*(III)*
**S3**	E	*CantonS*
**S3**	F	*Clock*^{TIM}*(III)*
**S3**	G	*esg-Gal4/UAS-mCherry;ClockTIM/MKRS or TM6B (for quantification and left image panel) also esg-Gal4,UAS-mCherry,Clock*^{TIM}*/CyO;tubGal80*^{TS}*/TM6B*
**S3**	H	*Myo1A-Gal4/UAS-mCherry;ClockTIM/MKRS or TM6B*

**S3**	I	*esg-Gal4,Clock^{TIM},UAS-mCherry/+;ry^{506}/TM6B; esg-Gal4,Clock^{TIM},UAS-mCherry/IF or CyO;cyc^{0}, ry^{506}; esg-Gal4,Clock^{TIM},UAS-mCherry/UAS-cyc; cyc^{0}, ry^{506}*
**S4**	A	*Clock^{TIM}(III)*
**S4**	B	*Clock^{PER}(II) and Clock^{TIM}(III)*
**S4**	C	*Clock^{TIM}(III)*
**S5**	A-D	*CantonS*
**S6**	A	*Clock^{TIM};Pdp1^{3135}*
**S6**	B-C	*CantonS*
**S6**	D	*act>y+>Gal4,UAS-CD2/hs-Flp;Clock^{TIM}/+*
**S6**	E	*act>y+>Gal4,UAS-CD2/hs-Flp;Clock^{PER}/+*
**S7**	A-E	*Clock^{TIM}(III)*

Statistics

Figure	Panel	Test	Details
**1**	H	One-Way ANOVA	Clk: F=82.47, p<0.0001; tim: F=17.34, p=0.0001; Pdp1: F=15.97, p=0.0001; vri: F=24.03, p<0.0001
**2**	A	One-Way ANOVA<br><br><br><br><br><br><br><br>Two-Way ANOVA	Day 1: Pdp1: F=1.478, p=0.2969; vri: F=1.961, p=0.1827; cry: F=1.899, p=0.1942; tim: F=2.471, p=0.1142; per: F=9.651, p=0.0024; Clk: F=2.699,p=0.0939; cyc F=4.366, p=0.0277<br>Day 4: Pdp1: F=5.958, p=0.0112; vri: F=1.946, p=0.1853; cry: F=2.287, p=0.1346; tim: F=4.000, p=0.0352; per: F=4.082, p=0.0333; Clk: F=0.3662, p=0.8983; cyc F=0.5829, p=0.7544<br>Pdp1: F=1.141, p=0.3867; vri: F=1.421, p=0.2637; cry: F=3.156, p=0.0270; tim: F=0.9139, p=0.5207; per: F=1.422, p=0.2634; Clk: F=0.8850, p=0.5399; cyc F=0.9447, p=0.5007
**2**	B	One-Way ANOVA<br><br>Cosinor	Clock^{PER}: F=7.721, p<0.0001, Day 1 F=5.611, p<0.0001, Day 4 F=10.71, p<0.0001<br>Day 1: rhythmic p-value 0.31, amplitude 0.19, peak time 19.3.<br>Day 4: rhythmic p-value 2.86E-6, amplitude 0.5, peak time 2.9.
**2**	C	One-Way ANOVA<br><br>Cosinor	Clock^{TIM}: F=15.07, p<0.0001, Day 1 F=6.170, p<0001, Day 4 F=14.93, p<0.0001<br>Day 1: rhythmic p-value 3.03E-8, amplitude 0.34, peak time 3.6.<br>Day 4: rhythmic p-value 1.16E-16, amplitude 0.37, peak time 22.5<br>Day 1 vs. Day 4: mesor difference -0.53, p-value 4.92E-24; amplitude difference 0.34, p-value 0.65; phase difference -5.93E-12
**2**	D	One-Way ANOVA	F=6.440, p<0.0001

**3**	C-D	Pearson's Correlation Matrix	Shown below
**5**	A	One-Way ANOVA	F=4.646, p<0.0001, Holms-Sidac Multiple Comparisons, Luc vs.<br>Ecr#2 p<0.0001, Eip75B p=0.0154, Eip78C p<0.0001, gce p=0.0052, Hnf4 #1 p<0.0001, Hnf4 #2 p<0.0001, Hr3 #2 p=0.0040, Hr38 p<0.0001, Hr4 #1 p<0.0001, tai p=0.0031, usp #1 p=0.0288, usp #2 p=0.0030, Hr39 p<0.0001, Hr78 p=0.0001
**5**	B	One-Way ANOVA	F=3.995, p<0.0001, Holms-Sidac Multiple Comparisons, Control vs. Hnf4 #1 p=0.0355, Hr78 p=0.0127
**5**	C	One-Way ANOVA	F=6.818, p<0.0001, Holms-Sidac Multiple Comparisons, Luc vs.<br>EcR #1 p=0.0145, Eip75B #1 p=0.195, Eip75B #2 p=0.0003, ERR p=0.0017, Hnf4 #1 p<0.0001, Hnf4 #2 p=0.0001, Hr38 p=0.0017, Hr4#1 p<0.0001, Hr4 #2 p<0.0001, tai p=0.0017, usp#1 p=0.303, usp#2 p<0.0001
**6**	C	Kruskal-Wallis Test	RpL32: 494.7, p<0.0001; klu: 588.8, p<0.0001; Amy-p: 408.3, p<0.0001; Pdp1: 413.8, p<0.0001; tim: 294.7, p<0.0001; vri: 455.6, p<0.0001; cwo: 431.5, p<0.0001
**7**	A	One-Way ANOVA	F=52.19, p<0.0001
**7**	B	One-Way ANOVA	F=32.78, p<0.0001
**7**	C	Two-Way ANOVA	Feed/Starve: 3.037, p=0.0047; RF5-11: F=5.036, p<0.0001; RF0-6 F=12.21, p<0.0001
**S2**	B	Two-Way ANOVA	Area: F=0.6697, p=0.7808; Cell Density: F=0.9453, p=0.4970; AMPs/PCs: F=1.086, p=0.3658
**S2**	F	One-Way ANOVA	Day 1 Area: F=56.06, p<0.0001; Day 4 Area: F=27.84, p<0.0001
**S2**	G	One-Way ANOVA	Day1 Fluorescence: F=3.225, p=0.0304; Day 4 Fluorescence: F=0.7891, p=0.5062
**S3**	E	One-Way ANOVA	dbt: F=3.447, p=0.0569; cyc: F=9.053, p=0.0017; per: F=8.130, p=0.0021; cry: F=12.54, p=0.0004
**S3**	G	Mann-Whitney Test	p=0.4348, median of adult: 9.060, median of pupa: 2.730
**S3**	H	Mann-Whitney Test	p=0.0048, median adult: 38.18, median of pupa 19.95
**S4**	B	Cosinor Analysis	*Clock*^{PER}<br>Day 1-2: rhythmic p-value 0.00057, mesor 2.19, amplitude 0.38, peak time 3.64; Day 4-5: rhythmic p-value 5.47E-12, mesor 2.01, amplitude 0.56, peak time 1.68; Day 1-2 vs. 4-5: mesor difference -0.18, p-value 0.050; amplitude difference 0.17, p-value 0.18; phase difference -1.97, p-value 0.08

			Clock^{TIM}<br>Day1-2: rhythmic p-value 1.96E-16, mesor 1.6, amplitude 0.44, peak time 2.02; Day 4-5: rhythmic p-value 5.87E-18, mesor 1.29, amplitude 0.57, peak time 21.3; Day 1-2 vs. 4-5: mesor difference 1.26E-07, amplitude difference 0.13, p-value 0.09; phase difference -4.73, p-value 8.54E-14
**S4**	C	One-Way ANOVA	69.49, p<0.0001
**S6**	C	Kruskal-Wallis Test	Hnf4: 279.9, p<0.0001; Eip75B: 418.4, p<0.0001; gce: 448.7, p<0.0001; ftz-f1: 333.6, p<0.0001; EcR: 511.3, p<0.0001
**S7**	A	Two-Way ANOVA	F=9.094, p<0.0001
**S7**	C	Two-Way ANOVA	F=1.237, p=0.2834
**S7**	D	Two-Way ANOVA	F=3.224, p=0.0029
**S7**	E	Two-Way ANOVA	F=6.082, p<0.0001

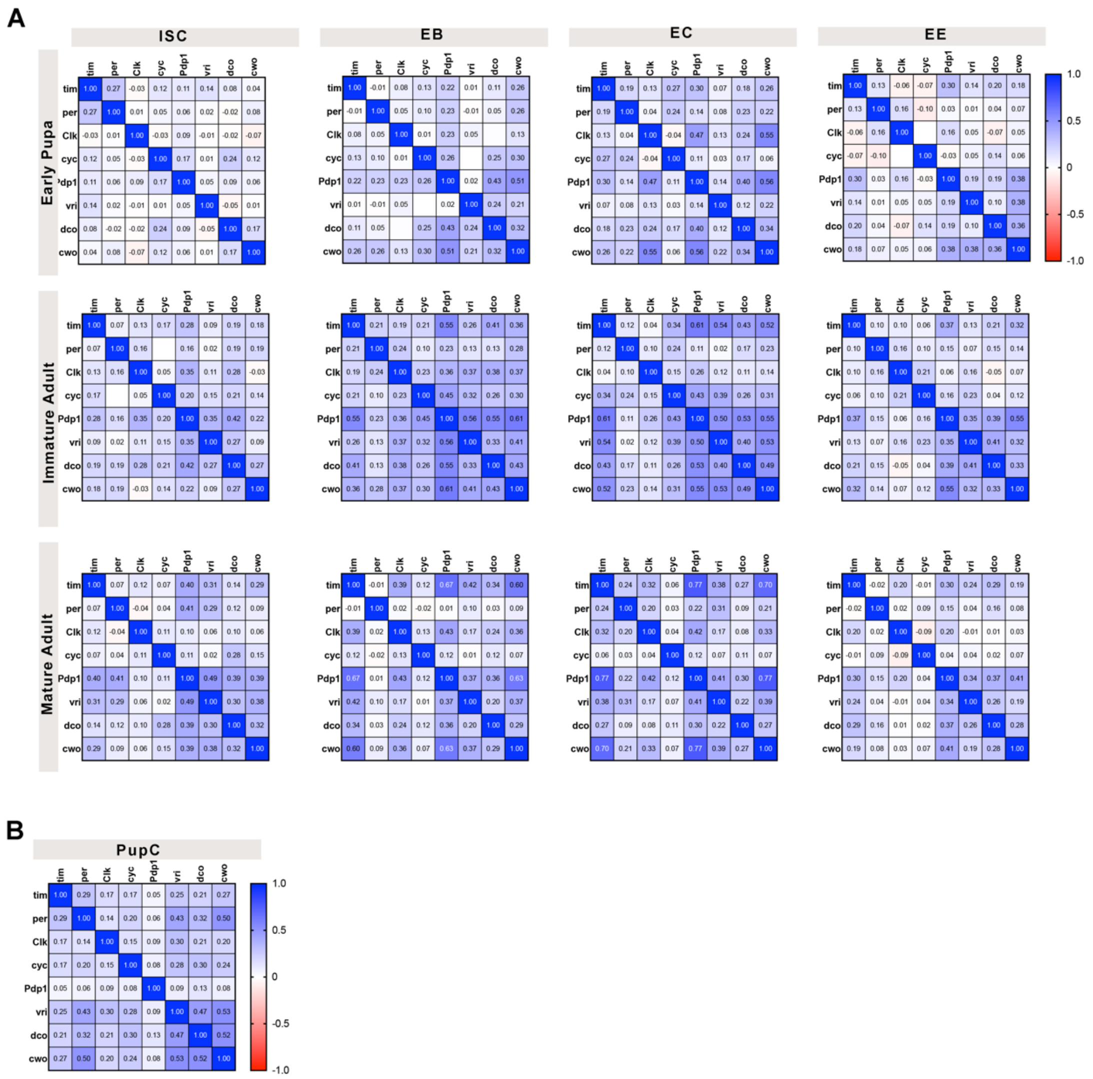

Supplementary Figure 1. Related to Figure 3. Correlation Matrices for Figure 3C-D. **(A)** Shows the correlation for each cell type separated by development stage. **(B)** Shows the correlation for all pupal cells (PupCs).

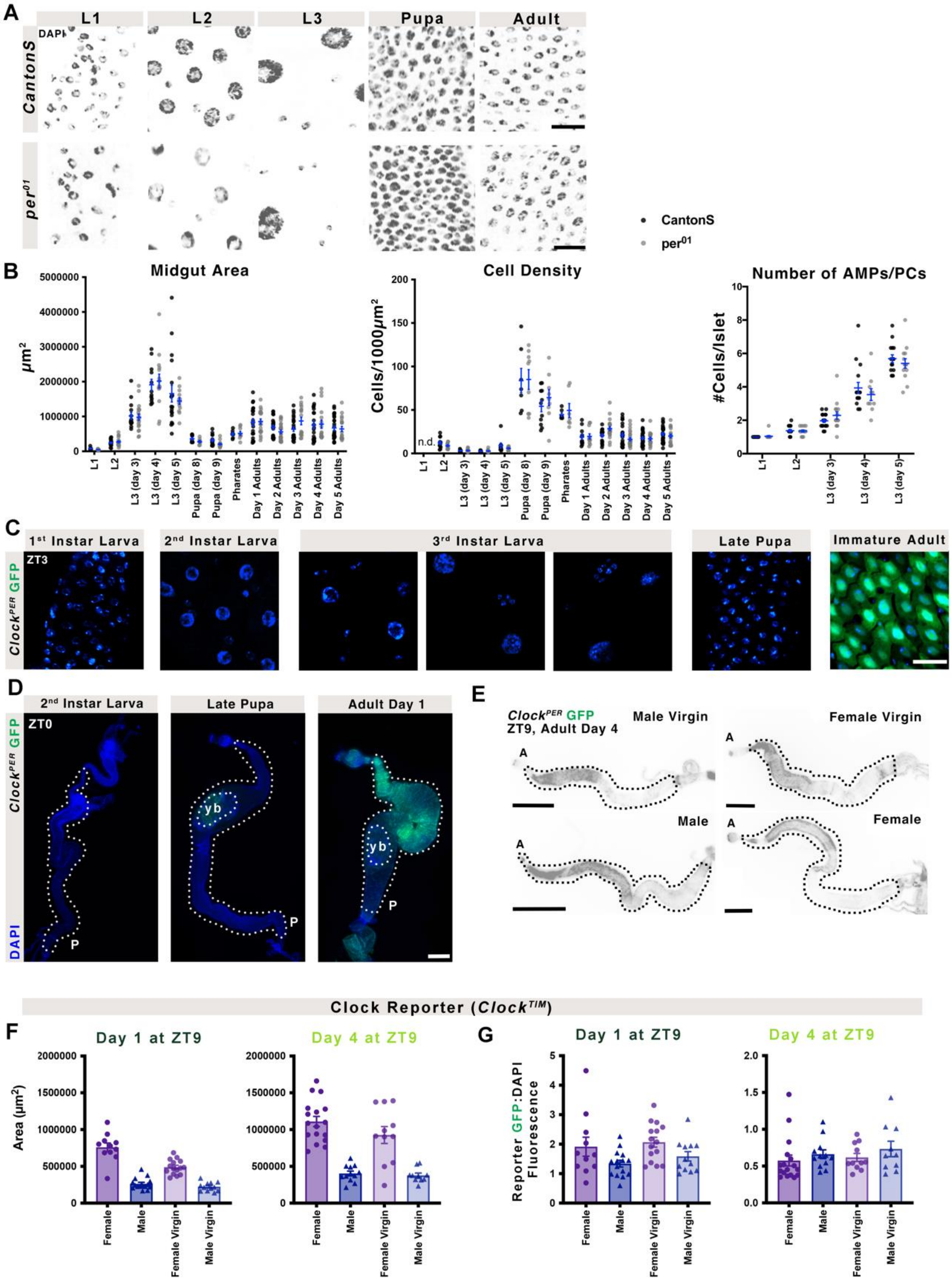
A
L1
L2
L3
Pupa
Adult
DAPI
CantonS
per01
CantonS
per01
B
Midgut Area
Cell Density
Number of AMPs/PCs
μm2
Cells/1000μm2
#Cells/Islet
n.d.
L1
L2
L3 (day 3)
L3 (day 4)
L3 (day 5)
Pupa (day 8)
Pupa (day 9)
Pharates
Day 1 Adults
Day 2 Adults
Day 3 Adults
Day 4 Adults
Day 5 Adults
C
1st Instar Larva
2nd Instar Larva
3rd Instar Larva
Late Pupa
Immature Adult
ZT3
ClockPER GFP
D
2nd Instar Larva
Late Pupa
Adult Day 1
ZT0
ClockPER GFP
DAPI
yb
P
E
ClockPER GFP
ZT9, Adult Day 4
Male Virgin
Female Virgin
Male
Female
A
Clock Reporter (ClockTIM)
F
Day 1 at ZT9
Day 4 at ZT9
Area (μm2)
G
Day 1 at ZT9
Day 4 at ZT9
Reporter GFP:DAPI Fluorescence
Female
Male
Female Virgin
Male Virgin

Supplementary Figure 2. Related to Figure 1. per^{01} and CantonS Flies Develop at the Same Rate. **(A)** *CantonS* and per^{01} representative images from larval stages (L1, L2, L3), pupa and adults. Images taken from the posterior intestine. Scale bar 20µm. DAPI stains nuclei. **(B)** Graphs show the total area of the intestine and the cell density in the posterior intestine. The number of cells per islet shows the adult midgut precursor cells (AMPs) and their niche forming peripheral cells (PCs) from L1 to L3. Each point represents one intestine. There are no significant differences between *CantonS* and per^{01}. **(C)** Representative images from $Clock^{PER}$ larval stages (L1, L2, L3), pupa and immature adult. Images taken from the posterior intestine showing Clk/cyc activity begins abruptly in the immature adult. Scale bar 20µm. DAPI stains nuclei. **(D)** Images of the $Clock^{PER}$ reporter intestine in 2nd instar, late pupa and adults showing Clk/cyc activity only in the adults. DAPI marks nuclei. Scale 200µm. “yb” indicates yellow body. “P” indicates posterior. **(E)** Representative images of $Clock^{PER}$ male, female and virgin intestines show similar patterns in reporter activity. Scale 500µm. “A” indicates anterior. **(F)** Male and male virgin flies are significantly smaller than female and female virgin flies at both day 1 and day 4 after pupation. One-Way ANOVA $p<0.0001$. **(G)** Males show slightly lower $Clock^{TIM}$ activity than females and female virgins on Day 1 but by Day 4 $Clock^{TIM}$ expression is not significantly different. Each point represents one intestine. One-Way ANOVA Day 1 $p=0.0304$. For graphs: Line/Bar shows mean, error bars indicate ±SEM, full statistics are shown in Supplementary Information. Representative images of two replicates. Source data are provided as a Source Data file.

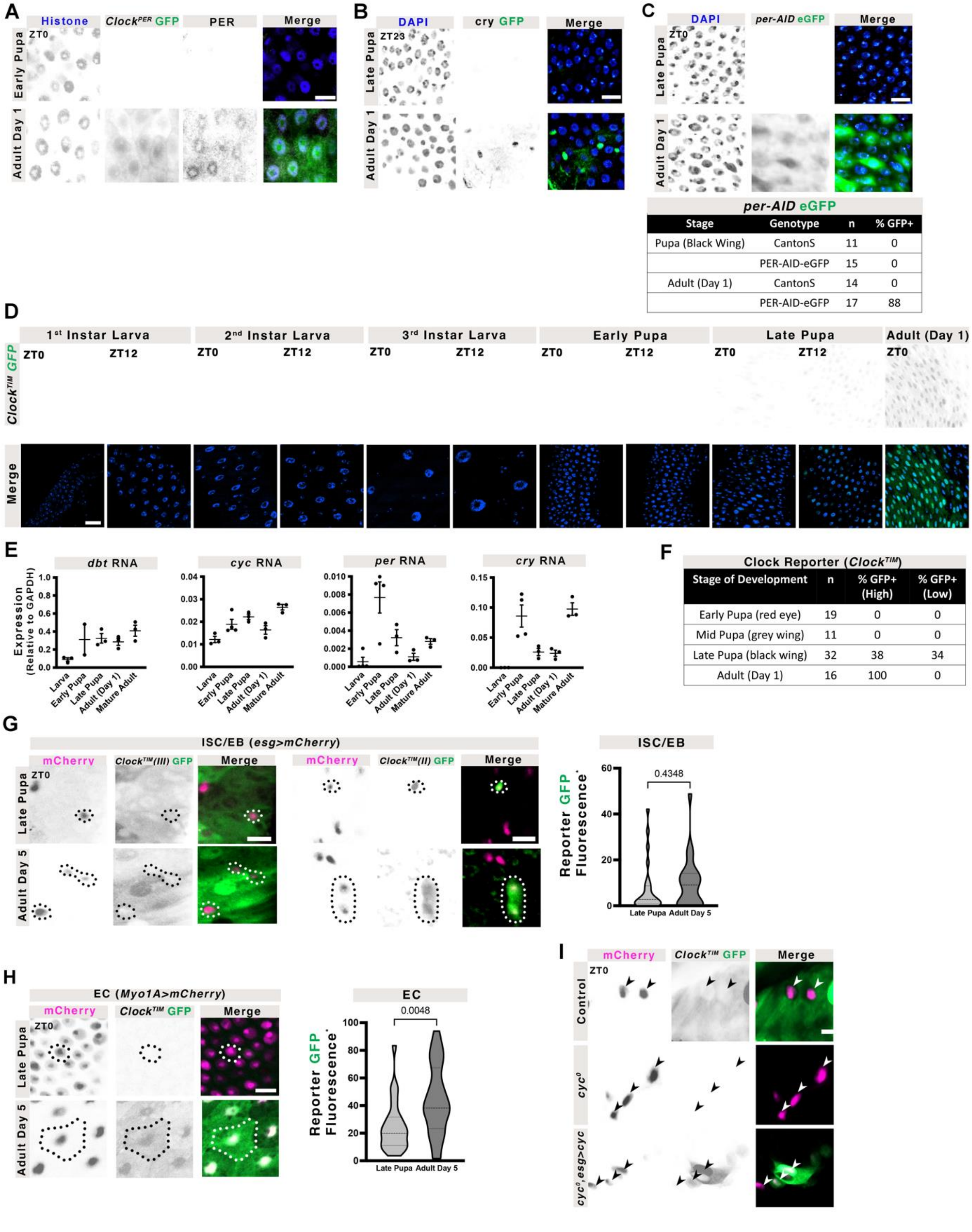

per-AID eGFP

Stage	Genotype	n	% GFP+
Pupa (Black Wing)	CantonS	11	0
	PER-AID-eGFP	15	0
Adult (Day 1)	CantonS	14	0
	PER-AID-eGFP	17	88

Clock Reporter (*Clock*^{TIM})

Stage of Development	n	% GFP+ (High)	% GFP+ (Low)
Early Pupa (red eye)	19	0	0
Mid Pupa (grey wing)	11	0	0
Late Pupa (black wing)	32	38	34
Adult (Day 1)	16	100	0

Supplementary Figure 3. Related to Figure 1. Circadian Clock Activity is present in Adults. **(A)** *Clock^{PER}* with PER antibody staining, **(B)** *cry-GFP*, **(C)** *PER-AID* flies (table shows *per-AID* quantification compared to non-fluorescent control) from late-stage pupa show clock protein expression only in the immature adult intestine after pupation is complete. Histone or DAPI stains nuclei. Scale bar 10µm. **(D)** Images of Clk/cyc activity at ZT0 and ZT12 from larva to adult indicate that clock activity during adults is not present during the day. Scale 20µm. DAPI marks nuclei. **(E)** RT-qPCR analysis show that *dbt* and *cyc* are expressed throughout development, whereas *per* and *cry* show low levels in larva but higher levels in early pupa and adults. Error bars indicate ±SEM. Each point represents one replicate (20 intestines). One-Way ANOVA $p<0.05$ except for *dbt* $p=0.0569$, full Statistics in Supplementary Information. **(F)** Quantification of the *Clock^{TIM}* reporter throughout pupation, from dissected intestines (compare to whole mount quantification shown in Figure 1). This confirms that the expression is much lower in the late stage pupa than the immature adult, with intestines exhibiting Clk/cyc activity at the pupa-to-adult transition. **(G)** Clk/cyc activity in ISC/EBs (marked by *mCherry* expression in *esg+* cells, representative cells are outlined) is similar in late pupa and immature adults, Mann-Whitney test p-values are shown on the graph. Two reporters are shown, the quantification corresponds to *Clock^{TIM}* (III) but due to its weaker expression in ISC/EBs, we have included an image of *Clock^{TIM}* (II) which shows a similar pattern but stronger GFP expression in ISC/EBs. **(H)** In contrast, ECs (*Myo1A>mCherry*) have significantly higher Clk/cyc activity in adults. Together these data suggest that ISC/EB clocks are established earlier in intestinal development, but EC clocks are established later. Scale 10µm. A representative EC is outlined. Mann-Whitney test p-values are shown on the graph. **(I)** Representative images of *Clock^{TIM}* intestines showing that the Clk/cyc activity that is present in the controls, is absent in the *cyc^{01}* mutant, and restored strongly in the ISC/EBs when cyc is overexpressed (*esg>mCherry, cyc*). Arrowheads indicate mCherry+ ISC/EBs. Scale bar 10µm. Full statistics are shown in Supplementary Information. Representative images of two replicates. Source data are provided as a Source Data file.

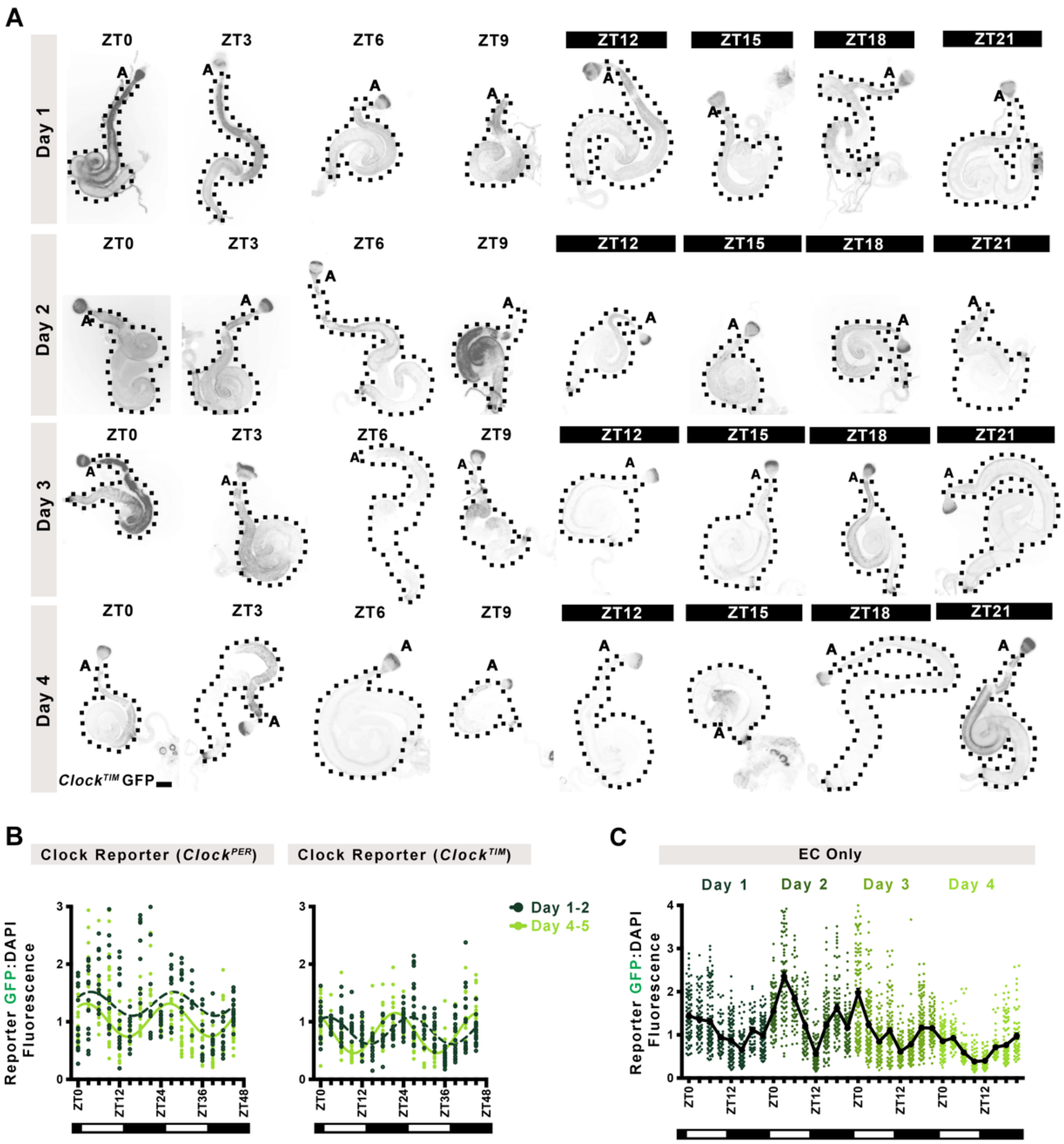

Supplementary Figure 4. Related to Figure 2. Clk/cyc Activity is Rhythmic at Adult Day 4 in ECs. **(A)** Representative images of Clk/cyc (*Clock*^{TIM}) activity over the first four days in adult flies, showing rhythms are stochastic on days 1-2 and gradually become established at day 4 (maximum at ZT0 and minimum at ZT12) [1]. Of note, GFP+ expression is weaker in the central region compared to the anterior and posterior regions at all times. Scale bar 200µm. "A" indicates anterior. **(B)** Cosinor analyses for days 1-2 compared with 4-5 for *Clock*^{PER} and *Clock*^{TIM} show slight shifts in the peak time during early clock activity. We interpret these with caution, however, since the intestine undergoes developmental maturation and may be dissimilar in the differentiated cell population on day 1 compared with day 2. **(C)** ECs show similar patterns in clock development as the whole intestine (compare to Figure 2C). Each point represents one EC, n=3115. One-Way ANOVA p<0.0001. For graphs: Line shows mean, error bars indicate ±SEM, full statistics are shown in Supplementary Information. Representative images of two replicates. Source data are provided as a Source Data file.

A

Summary of Cell Groups

Cell Type	Cluster Numbers	% Total Cells	% Pupal Cells	% Immature Adult Cells	% Adult Cells
ISC	0	14.20%	13.02%	20.80%	11.41%
EB	2,3	40.81%	11.43%	18.30%	26.36%
EE	5,11,16,21	11.39%	10.63%	22.47%	5.45%
EC	4,6,7,8,9,10,12,14,17, 18,19,20,22	40.81%	30.70%	32.00%	56.41%
PupC	1,13	12.97%	30.25%	5.18%	0.10%
VM	15	1.93%	3.97%	1.25%	0.25%
**Total**	23 clusters	5190 cells	2013 cells	1197 cells	1980 cells

B

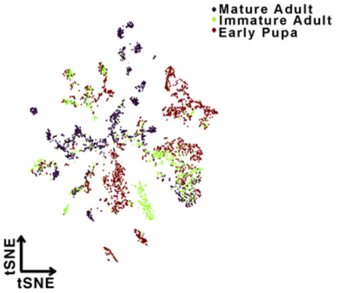

C

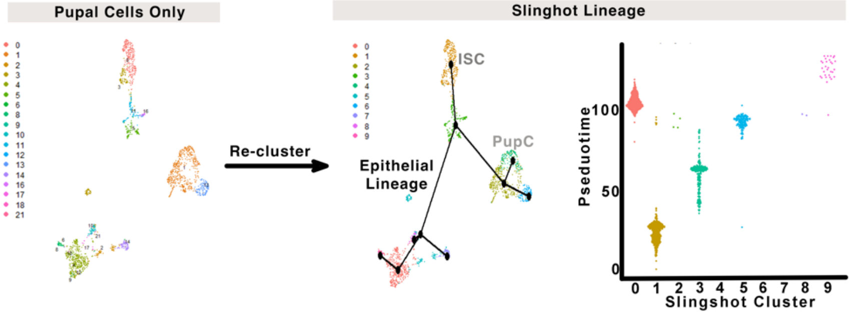

D

Early Pupa

Gene	Average RNA Count	%Cells With No Gene Expression
tim	0.35	77%
per	0.17	87%
Clk	0.10	93%
cyc	0.39	74%
Pdp1	3.02	44%
vri	0.86	71%
dbt	0.81	60%
cwo	1.90	50%

Immature Adults

Gene	Average RNA Count	%Cells With No Gene Expression
tim	1.09	54%
per	0.17	87%
Clk	0.27	84%
cyc	0.39	75%
Pdp1	11.80	21%
vri	1.32	53%
dbt	1.46	46%
cwo	2.92	45%

Mature Adults

Gene	Average RNA Count	%Cells With No Gene Expression
tim	1.90	42%
per	0.15	90%
Clk	0.15	89%
cyc	0.15	88%
Pdp1	10.51	17%
vri	0.92	64%
dbt	0.74	65%
cwo	1.90	44%

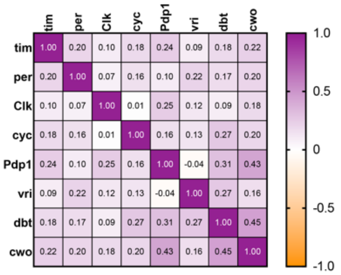

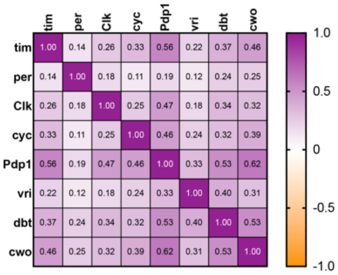

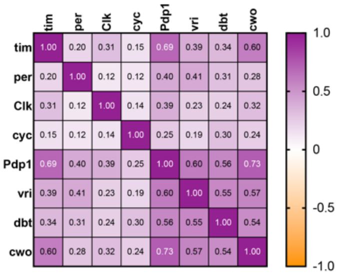

Supplementary Figure 5. Related to Figure 3. Adult Cells Express Circadian Clock Genes at Higher Levels. **(A)** The cell types and the proportion of cells that were collected during scRNAseq. The ISCs, EBs, ECs, EEs and VM were recovered at all three developmental stages whereas PupCs were only present in the pupa and adults. **(B)** The tSNE plot shows the overlap between the three datasets after integration. **(C)** UMAP for the pupal cells only, without VM, with predicted lineages demonstrates that PupCs form a distinct state separate from the epithelial lineage derived from ISC/AMPs. **(D)** Chart shows the average counts detected for a set of circadian clock genes, the genes with highest detection were used for further analysis. Pearson's correlation matrix shows the clock genes *tim, Pdp1, vri,* and *cwo* are positively correlated more strongly in mature adult intestines than at earlier stages.

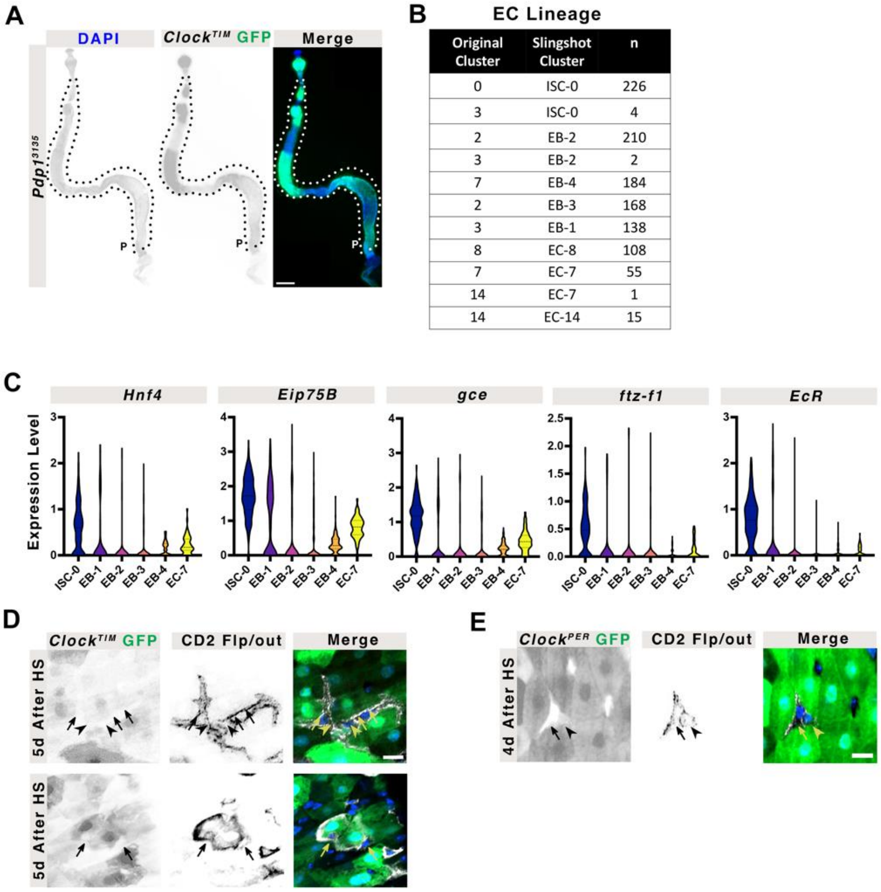

EC Lineage

Original Cluster	Slingshot Cluster	n
0	ISC-0	226
3	ISC-0	4
2	EB-2	210
3	EB-2	2
7	EB-4	184
2	EB-3	168
3	EB-1	138
8	EC-8	108
7	EC-7	55
14	EC-7	1
14	EC-14	15

Supplementary Figure 6. Related to Figure 4-6. Testing the Correlation of Circadian Clock Expression and ISC Differentiation. **(A)** Mutants of the isoform of Pdp1 responsible for circadian rhythms (*Pdp1*^{3135}) show Clk/cyc activity after eclosion, suggesting that loss of Pdp1 is not sufficient to inhibit circadian clock development. **(B)** EC lineage numbers correspond to the original clusters, the number of cells after re-clustering are presented in the table. These were further analyzed in Figure 6. **(C)** Nuclear receptor expression during EC differentiation shows a similar pattern to clock genes during differentiation (*Hnf4, Eip75B, gce*) and may influence this change in clock activity. Lines indicate median and quartiles. **(D)** Representative images of CD2-labeled ISC clones with *Clock*^{TIM} expression heterogenous in the population of small cells spawned from ISCs. Similarly a *Clock*^{PER} clone **(E)** is shown. DAPI marks nuclei. Scale bar 10µm. Arrowheads mark GFP+ cells, arrows mark GFP- cells. Representative images of two replicates.

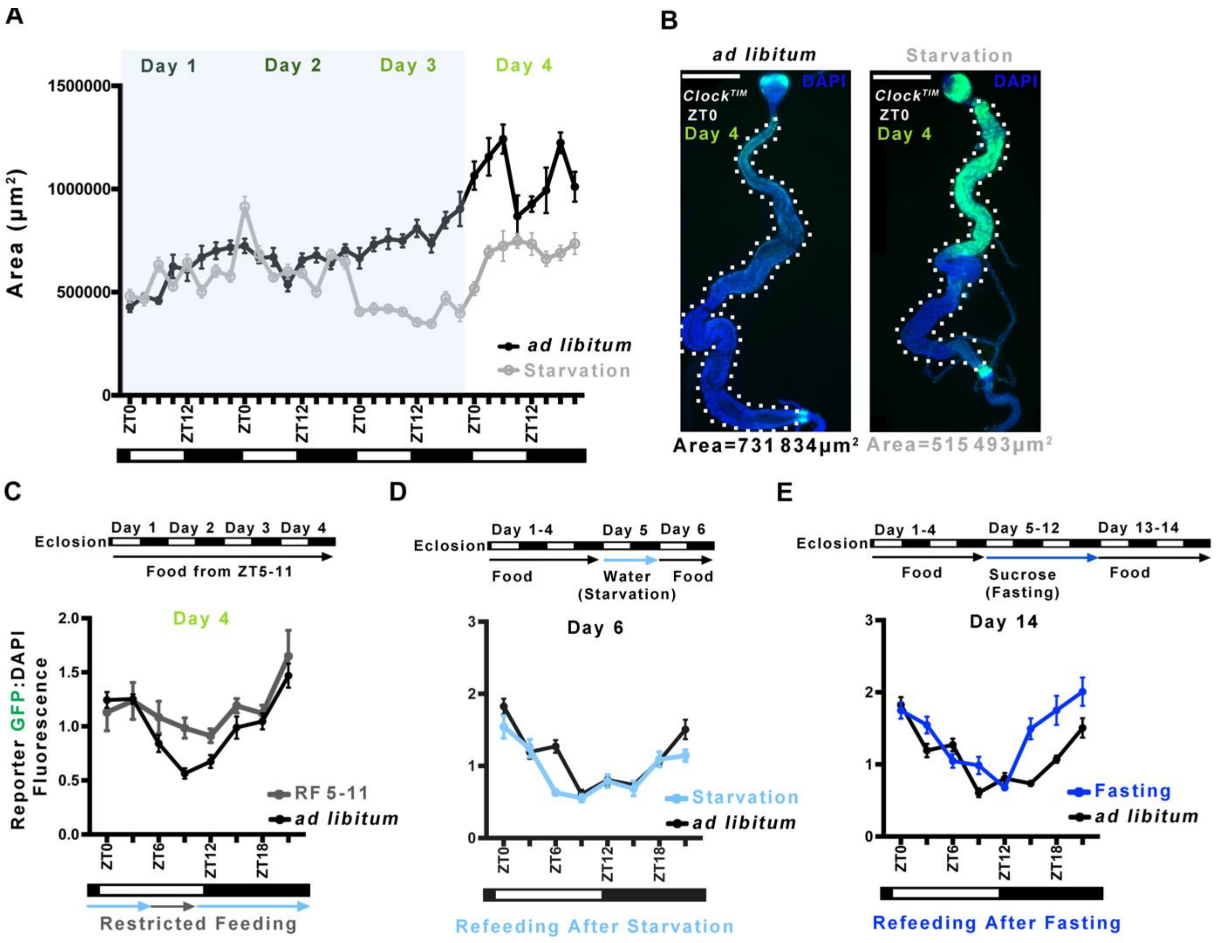

Supplementary Figure 7. Related to Figure 7. Fasting Does Not Disrupt Established Rhythms. **(A-B)** Fasting for the first 3 days after pupation decreases the size of the adult intestine, however does not affect Clk/cyc (*Clock*^{TIM}) activity (compare *ad libitum*-fed (Figure 2C) vs. starvation conditions Figure 7A). DAPI stains nuclei. Scale bar 500µm. **(C)** Restricted feeding of control *Clock*^{TIM} reporter flies does not change daily rhythms, in contrast to the same reporter in *cry* mutants (compare to Figure 7C). Since *cry* mutants cannot transduce photoperiod light to the circadian clock system, this suggests photoperiod is dominant over restricted feeding to synchronize the maturing intestinal clock. Two-way ANOVA p-value=0.2834. **(D)** Starvation (one day water only) and **(E)** fasting (one week sucrose only diet) do not significantly change circadian clock transcription. In both cases, Clk/cyc activity is rhythmic, with a peak around ZT0 and trough around ZT12. Control (black) shows same data from control-fed (*ad libitum*) flies on both D-E graphs. Two-Way ANOVA $p<0.05$. For graphs: Line shows mean, error bars indicate ±SEM, full statistics are in Supplementary Information. Representative images of two replicates. Source data are provided as a Source Data file.

References

1. Parasram, K., et al., *Intestinal Stem Cells Exhibit Conditional Circadian Clock Function.* Stem cell reports, 2018. **11**(5): p. 1287-1301.
2. Bainbridge, S.P. and M. Bownes, *Staging the metamorphosis of Drosophila melanogaster.* Development, 1981. **66**(1): p. 57-80.
3. Karpowicz, P., et al., *The circadian clock gates the intestinal stem cell regenerative state.* Cell reports, 2013. **3**(4): p. 996-1004.
4. Bu, B., et al., *Nipped-A regulates the Drosophila circadian clock via histone deubiquitination.* The EMBO Journal, 2020. **39**(1).
5. Benito, J., et al., *The blue-light photoreceptor CRYPTOCHROME is expressed in a subset of circadian oscillator neurons in the Drosophila CNS.* Journal of biological rhythms, 2008. **23**(4): p. 296-307.
