# Supplementary material for: The emergence of circadian timekeeping in the intestine
Source: Nat Commun. 2024 Feb 27;15:1788. doi: 10.1038/s41467-024-45942-4 (PMC10899604; doi:10.1038/s41467-024-45942-4)
Supplement: Supplementary file 3 — Description of Additional Supplementary Files [file 41467_2024_45942_MOESM3_ESM.pdf]

Description of Additional Supplementary Files

File Name: Supplementary Data 1
Description: Related to Figure 2. Early Pupa Intestine Raw Counts.

File Name: Supplementary Data 2
Description: Related to Figure 2. Immature Adult Intestine Raw Counts.

File Name: Supplementary Data 3
Description: Related to Figure 2. Mature Adult Intestine Raw Counts.

File Name: Supplementary Data 4
Description: Related to Figure 3-6. Cluster Markers, determined by Seurat using ROC analysis with a logFC threshold of 0.25 and minimum percentage of cells of 0.25.

File Name: Supplementary Data 5
Description: Related to Figure 5. ClockTIM RNAi Screen.
