# Supplementary material for: The emergence of circadian timekeeping in the intestine
Source: Nat Commun. 2024 Feb 27;15:1788. doi: 10.1038/s41467-024-45942-4 (PMC10899604; doi:10.1038/s41467-024-45942-4)
Supplement: Supplementary file 9 — Reporting Summary [file 41467_2024_45942_MOESM9_ESM.pdf]

nature portfolio

Corresponding author(s): Phillip Karpowicz

Last updated by author(s): Jan 29, 2024

Reporting Summary

Nature Portfolio wishes to improve the reproducibility of the work that we publish. This form provides structure for consistency and transparency in reporting. For further information on Nature Portfolio policies, see our Editorial Policies and the Editorial Policy Checklist.

Statistics

For all statistical analyses, confirm that the following items are present in the figure legend, table legend, main text, or Methods section.

n/a	Confirmed	
☐	☒	The exact sample size (*n*) for each experimental group/condition, given as a discrete number and unit of measurement
☐	☒	A statement on whether measurements were taken from distinct samples or whether the same sample was measured repeatedly
☐	☒	The statistical test(s) used AND whether they are one- or two-sided<br>*Only common tests should be described solely by name; describe more complex techniques in the Methods section.*
☐	☒	A description of all covariates tested
☐	☒	A description of any assumptions or corrections, such as tests of normality and adjustment for multiple comparisons
☐	☒	A full description of the statistical parameters including central tendency (e.g. means) or other basic estimates (e.g. regression coefficient) AND variation (e.g. standard deviation) or associated estimates of uncertainty (e.g. confidence intervals)
☒	☐	For null hypothesis testing, the test statistic (e.g. *F*, *t*, *r*) with confidence intervals, effect sizes, degrees of freedom and *P* value noted<br>*Give P values as exact values whenever suitable.*
☒	☐	For Bayesian analysis, information on the choice of priors and Markov chain Monte Carlo settings
☒	☐	For hierarchical and complex designs, identification of the appropriate level for tests and full reporting of outcomes
☒	☐	Estimates of effect sizes (e.g. Cohen's *d*, Pearson's *r*), indicating how they were calculated

Our web collection on statistics for biologists contains articles on many of the points above.

Software and code

Policy information about availability of computer code

Data collection	Data collection was not performed using code.
Data analysis	Photoshop (23.4.2, Adobe), Prism (v9, GraphPad), Cell Ranger (3.0.2, 10XGenomics), R (4.2.0), Slingshot (2.8.0), SCENIC (1.3.1), Seurat (4.3.0.1), ZenBlue (2.3, Zeiss)

For manuscripts utilizing custom algorithms or software that are central to the research but not yet described in published literature, software must be made available to editors and reviewers. We strongly encourage code deposition in a community repository (e.g. GitHub). See the Nature Portfolio guidelines for submitting code & software for further information.

Data

Policy information about availability of data

All manuscripts must include a data availability statement. This statement should provide the following information, where applicable:

- Accession codes, unique identifiers, or web links for publicly available datasets
- A description of any restrictions on data availability
- For clinical datasets or third party data, please ensure that the statement adheres to our policy

The scRNA-seq data generated in this study have been deposited in the NCBI GEO database under accession codeGSE230572 [https://www.ncbi.nlm.nih.gov/geo/query/acc.cgi?acc=gse230572]. The alignments were made using Drosophila reference genome 6.25 and are publicly available as of the date of publication. Source data are provided with this paper.

Research involving human participants, their data, or biological material

Policy information about studies with human participants or human data. See also policy information about sex, gender (identity/presentation), and sexual orientation and race, ethnicity and racism.

Reporting on sex and gender	*Use the terms sex (biological attribute) and gender (shaped by social and cultural circumstances) carefully in order to avoid confusing both terms. Indicate if findings apply to only one sex or gender; describe whether sex and gender were considered in study design; whether sex and/or gender was determined based on self-reporting or assigned and methods used. Provide in the source data disaggregated sex and gender data, where this information has been collected, and if consent has been obtained for sharing of individual-level data; provide overall numbers in this Reporting Summary. Please state if this information has not been collected. Report sex- and gender-based analyses where performed, justify reasons for lack of sex- and gender-based analysis.*
Reporting on race, ethnicity, or other socially relevant groupings	*Please specify the socially constructed or socially relevant categorization variable(s) used in your manuscript and explain why they were used. Please note that such variables should not be used as proxies for other socially constructed/relevant variables (for example, race or ethnicity should not be used as a proxy for socioeconomic status). Provide clear definitions of the relevant terms used, how they were provided (by the participants/respondents, the researchers, or third parties), and the method(s) used to classify people into the different categories (e.g. self-report, census or administrative data, social media data, etc.) Please provide details about how you controlled for confounding variables in your analyses.*
Population characteristics	*Describe the covariate-relevant population characteristics of the human research participants (e.g. age, genotypic information, past and current diagnosis and treatment categories). If you filled out the behavioural & social sciences study design questions and have nothing to add here, write "See above."*
Recruitment	*Describe how participants were recruited. Outline any potential self-selection bias or other biases that may be present and how these are likely to impact results.*
Ethics oversight	*Identify the organization(s) that approved the study protocol.*

Note that full information on the approval of the study protocol must also be provided in the manuscript.

Field-specific reporting

Please select the one below that is the best fit for your research. If you are not sure, read the appropriate sections before making your selection.

☒ Life sciences ☐ Behavioural & social sciences ☐ Ecological, evolutionary & environmental sciences

For a reference copy of the document with all sections, see nature.com/documents/nr-reporting-summary-flat.pdf

Life sciences study design

All studies must disclose on these points even when the disclosure is negative.

Sample size	A minimum of n=3-5 samples is typically needed for our analyses, however, a sample size 7-30 was used to ensure biological reproducibility. In some cases, low sample viability resulted in larger variation.
Data exclusions	No data points were excluded from the analysis.
Replication	Experiments were successfully replicated 7-30 biological replicates and these were replicated on several different occasions (at least 2-3 times).
Randomization	Samples were pooled and randomly sorted prior to sampling.
Blinding	Researchers were not double-blinded due to logistical issues when collecting circadian samples, and this is not typically performed in Drosophila-based research.

Reporting for specific materials, systems and methods

We require information from authors about some types of materials, experimental systems and methods used in many studies. Here, indicate whether each material, system or method listed is relevant to your study. If you are not sure if a list item applies to your research, read the appropriate section before selecting a response.

Materials & experimental systems

n/a	Involved in the study	
☐	☒	Antibodies
☒	☐	Eukaryotic cell lines
☒	☐	Palaeontology and archaeology
☐	☒	Animals and other organisms
☒	☐	Clinical data
☒	☐	Dual use research of concern
☒	☐	Plants

Methods

n/a	Involved in the study	
☒	☐	ChIP-seq
☒	☐	Flow cytometry
☒	☐	MRI-based neuroimaging

Antibodies

Antibodies used	PER (gift from Patrick Emery, 1:1500), GFP (abcam, Ab290, 1:2000, lot#GR3321614-1), Histone (EMD Millipore, MABE71, 1:2000), CD2 (BioRad, MCA154GA, 1:2000, lot#148975), secondary antibodies (Invitrogen, 1:2000) were goat anti-mouse 594 (A-11032), goat anti-rabbit 488 (A-11034, lot#1616933), goat anti-rabbit 647 (A-21245,lot#1660844).
Validation	Antibodies were validated prior to use and have been used in previous publications including: PER and histone (Parasram et al. 2018), GFP (Agrawal et al. 2017).

Animals and other research organisms

Policy information about studies involving animals; ARRIVE guidelines recommended for reporting animal research, and Sex and Gender in Research

Laboratory animals	Drosophila melanogaster strains in this study include: ClockPER, ClockTIM described in (Parasram et al. 2018). From Jadwiga Giebultowicz: CantonS and per01(isogenic with CantonS). From Chrysoula Pitsouli esg-Gal4 (II), tubGal80TS(III). From Yong Zhang PER-AID-GFP. From Steve Jean Su(H)GBE-Gal80, tubGal80ts(III). From Amita Sehgal Pdp13135(III). From Bloomington: #38424 UAS-mCherry (III), UAS-mCherry(II), myo1A-Gal4 (II) , #42563 Valium20-cyc(II), cry01, #76317 CRY-GFP, #4412 act>y+>Gal4,UAS-CD2, RNAi lines: #50712 and #58286 EcR (#1 and #2), #43231 Eip75B, #26718 Eip78C, #61937 and #50868 ERR, #27659 ftz-f1, #61852 gce, #29375 and #64988 Hnf4, #51442 and #27254 Hr3 (#1 and #2), #29376 and #29377 Hr38, #33624 and #27086 Hr39, #54803 and #31868 Hr4, #31990 Hr78, #31603 Luciferase, #26205 met, #36095 tai, #36729 and #27258 Usp (#1 and #2). From FlyORF: F001915 gce, F000656 Eip78C, F000144 and F003392 Hnf4 (#1 and #2), F000423 and F003303 Hr78 (#1 and #2), F004847 and F002207 Eip75B (#1 and #2), F003474 met. Flies were balanced with yw;IF/CyO;MKRS/TM6B. Age ranges from first larval instar to adults (day 1-14).
Wild animals	This study did not involve wild animals.
Reporting on sex	No differences were noted between females and males (S1E-G, Fig 5A,C), so females were primarily used in this study.
Field-collected samples	This study did not involve animals collected from the field.
Ethics oversight	No ethical approval or guidance was required for this study.

Note that full information on the approval of the study protocol must also be provided in the manuscript.
